# Mental health of children and young people with pre-existing eating problems during the COVID-19 pandemic

**DOI:** 10.1007/s40519-025-01788-3

**Published:** 2025-09-29

**Authors:** Johanna Lee, Natalia K. Rojas, Snehal M. Pinto Pereria, Terence Stephenson, Jennifer McGowan, Trudie Chalder, Emma Dalrymple, Tamsin Ford, Isobel Heyman, Shamez Ladhani, Kelsey McOwat, Ruth Simmons, Olivia Swann, CLoCk Consortium, Roz Shafran

**Affiliations:** 1 UCL GOS Institute of Child Health, London, England UK; 2 University College London Division of Surgery and Interventional Science, London, England UK; 3 UCL Department of Experimental Psychology, London, England UK; 4 King’s College London Department of Psychological Medicine, London, England UK; 5 Cambridge University, Psychiatry, Cambridge, England UK; 6 UK Health Security Agency, London, England UK; 7 The University of Edinburgh Usher Institute of Population Health Sciences and Informatics, Edinburgh, Scotland UK

**Keywords:** Children and young people, Eating problems, Mental health, COVID-19, Pandemic

## Abstract

**Objective:**

The study sought to explore mental health trajectories of children and young people (CYP) who retrospectively reported eating problems prior to the pandemic, over a 2-year period (2021–23). Given the rapid increase in eating disorder presentations during the pandemic, these CYP may be particularly susceptible to pandemic-related challenges, including social and functional restrictions.

**Methods:**

Data on 2023 CYP from the Children and Young People with Long COVID (CLoCk) study recruited Jan–March 2021 who completed questionnaires at 3-, 6-, 12-, and 24-months post SARS-CoV-2 PCR-testing were analysed. Associations between baseline eating problems (*N* = 241) and emotional and behavioural symptoms (measured by the Strengths and Difficulties Questionnaire (SDQ) total difficulties and impact scores) at each time-point were examined by regression models. Multi-level models were used to determine whether SDQ total and impact trajectories of those with/without prior self-reported eating problems differed.

**Results:**

Compared to CYP who did not report pre-existing eating problems, those that did had more mental health difficulties at all time points: reflected in significantly higher SDQ total difficulties and impact scores. However, mental health scores of CYP reporting pre-pandemic eating problems were stable over time. Whereas, CYP without eating problems had a slight increase in mental health difficulties over time. Differences between groups diminished but remained significant when controlling for potential confounding variables including prior mental health difficulties.

**Discussion:**

Young people with eating problems had more emotional and behavioural symptoms during 2021–23, compared with those that did not have eating problems. However, mental health did not worsen over time amongst CYP with pre-existing eating problems, providing evidence of some relative resilience to the effects of the pandemic in this population.

**Public significance:**

Eating disorders are a major public health concern and presentations have remained high since the Covid-19 pandemic. Understanding how eating difficulties relate to mental health symptomology over time has implications for service planning.

**Level of evidence:**

Level III: Evidence obtained from well-designed cohort study.

**Supplementary Information:**

The online version contains supplementary material available at 10.1007/s40519-025-01788-3.

## Introduction

Eating disorders are serious, debilitating psychiatric disorders characterised by persistent disturbances in eating behaviour, compensatory purging behaviours, and cognition that may reflect an individual’s distressing concerns about their body weight, shape, or image [1]. Eating disorders are clinically impairing and are diagnosed mental health conditions according to standardised diagnostic criteria, i.e. Diagnostic and Statistical Manual of Mental Disorders, Fifth Edition (DSM-V) [1] and/or International Classification of Diseases, 11th Revision (ICD-11) [2]. Subclinical eating disorders, often termed “eating problems” [3], are characterised by the experience of eating too little, eating too much, or eating in an uncontrolled manner (binge eating) but not reaching clinical threshold in terms of frequency, severity or impact. Such eating problems are relatively common and estimated at 22.4% in children and young people (CYP) aged between 11 and 17 years [4]. In England, subclinical eating disorders for CYP aged between 11 and 16 years increased from 6.7% in 2017 to 12.3% in 2023, and for CYP aged between 17 and 19 years, there was an increase from 44.6% in 2017 to 59.4% in 2023 [5]. Yet, it is important to note that not all difficulties with eating are indicative of an eating disorder per se. For example, some depressive symptoms reflect problems with eating, such as loss of appetite and weight loss or increased appetite and weight gain [1]. Additionally, there is significant comorbidity between eating disorders and other psychopathology, in particular depression and anxiety [6].

In times of uncertainty, young people with eating difficulties can struggle. They perceive uncertainty negatively, and in response to it, they report high levels of anxiety and stress, and may engage in a higher level of disordered eating behaviours to help regain control [7]. The Covid-19 pandemic created a context in which there was uncertainty. The spread of the SARS-CoV-2 virus disrupted the lives and experiences of individuals worldwide. As the virus spread, lockdown measures were imposed, and in the United Kingdom, the first national lockdown in March of 2020 ordered individuals to stay at home at all times except for essential purposes, such as shopping for groceries or medical appointments [8]. Over time, restrictions were eased and re-imposed as the waves of infection ran through the UK population, potentially leaving a large, long-term impact.

The mental health of CYP emerged as a major public health concern as research suggested detrimental effects of the pandemic [9–11], and those with pre-existing poor mental health difficulties such as anxiety or depression were more likely to report worse mental health compared to peers without difficulties in several studies [12–14]. CYP with pre-existing eating problems may have been prone to worsening eating and mental health difficulties during the pandemic for a variety of reasons, including social restrictions and lack of structure. CYP with eating disorders describe their social interactions outside of their family as vital during recovery from their eating disorders [15]. Losing access to their in-person social support networks during the pandemic may have been detrimental to these CYP’s mental health, possibly increasing anxiety and feelings of being socially isolated, which is particularly detrimental to CYP [16]. However, it could be that the lack of in-person socialisation reduced face-to-face body-based social comparisons, which may have been beneficial [17] although equally, online body-based social comparisons could have been detrimental. Moreover, people’s daily routines were disrupted due to school closures and the transition to remote work. Individuals with eating disorders expressed a loss of structure to their day—regarding their mealtimes and activities—as exacerbating their eating behaviours and mental health difficulties [18–21]. CYP with eating disorders similarly expressed concerns over the lack of routine [22].

There is a lack of research which has directly investigated the mental health trajectories over time for CYP with eating problems. In adults, Fernández-Aranda et al. [23] conducted a study investigating 29 women with eating disorders in the first 2 weeks of lockdown in Spain in early 2020 [23]. They found that 56.2% reported increased anxiety symptoms, including increased stress. Monteleone et al. [24] administered an online survey to 312 participants (mean age = 29.19) who had been diagnosed with an eating disorder and reported worsening psychopathology, including increased rates of anxiety and depression 6-months after initial easing of restrictions compared to prior to the pandemic [24]. Devoe et al. [25] conducted a systematic review of 53 studies on the impact of the Covid-19 pandemic on individuals with eating disorders, reporting an increase in anxiety and depressive symptoms amongst eating disorder patients [25]. However, to date, there is a lack of research which has investigated the long-term mental health trajectories of CYP with pre-existing eating difficulties during and after the COVID-19 pandemic.

### The present study

Our study aims to describe mental health trajectories of CYP with pre-existing eating problems during the aftermath of the pandemic. Our study proposes two research questions:

1. What are the associations between pre-existing eating problems and mental health of CYP 3-, 6-, 12- and 24-months post-recruitment into the CLoCk study?
2. Did CYP with pre-existing eating problems have different mental health trajectories over time compared to CYP without pre-existing eating problems?

Based on the previous literature [25], we hypothesised that CYP with retrospectively self-reported eating problems prior to the pandemic would have elevated mental health difficulties at all time points. We hypothesised that CYP with and without pre-existing eating problems would have differing mental health experiences over time, specifically that those with eating problems would have increased mental health difficulties over time compared to those without eating problems [23, 26].

## Methods

### The children and young people with long COVID (CLoCk) study

This study utilised data from the CLoCk study [27, 28], the largest cohort study of Long COVID in non-hospitalised CYP to date, which documented changes in the physical and mental health of over 30,000 CYP aged between 11 and 17 years across England. The primary aim of the CLoCk study was to analyse the long-term health trajectories of children/adolescents with a positive/negative PCR-test. In CLoCK, SARS-CoV-2 test-positive CYP aged between 11 and 17 years were matched on their age, sex at birth, region of residence, and month of testing to SARS-CoV-2 test-negative CYP identified by the United Kingdom Health Security Agency (UKHSA).

### Participants

The data analysed in this study was from consenting CYP who completed a PCR test between January and March 2021, and completed the CLoCk questionnaire at 3-, 6-, 12-, and 24-months post-testing. These correspond to April–June 2021 (3-months), July–September 2021 (6-months) and January–March 2022 and 2023 (12- and 24-months respectively) data collection time points.

Retrospectively self-reported eating problems were reported at enrolment into the study (April–June 2021). CYP were asked whether they had eating problems prior to the pandemic “such as eating too little, eating too much, or eating in an uncontrolled manner (binge eating)”. Accordingly, two groups were derived: (1) CYP who reported pre-existing eating problems prior to the pandemic; and (2) those who reported no pre-existing eating problems prior to the pandemic.

**Fig. 1:**
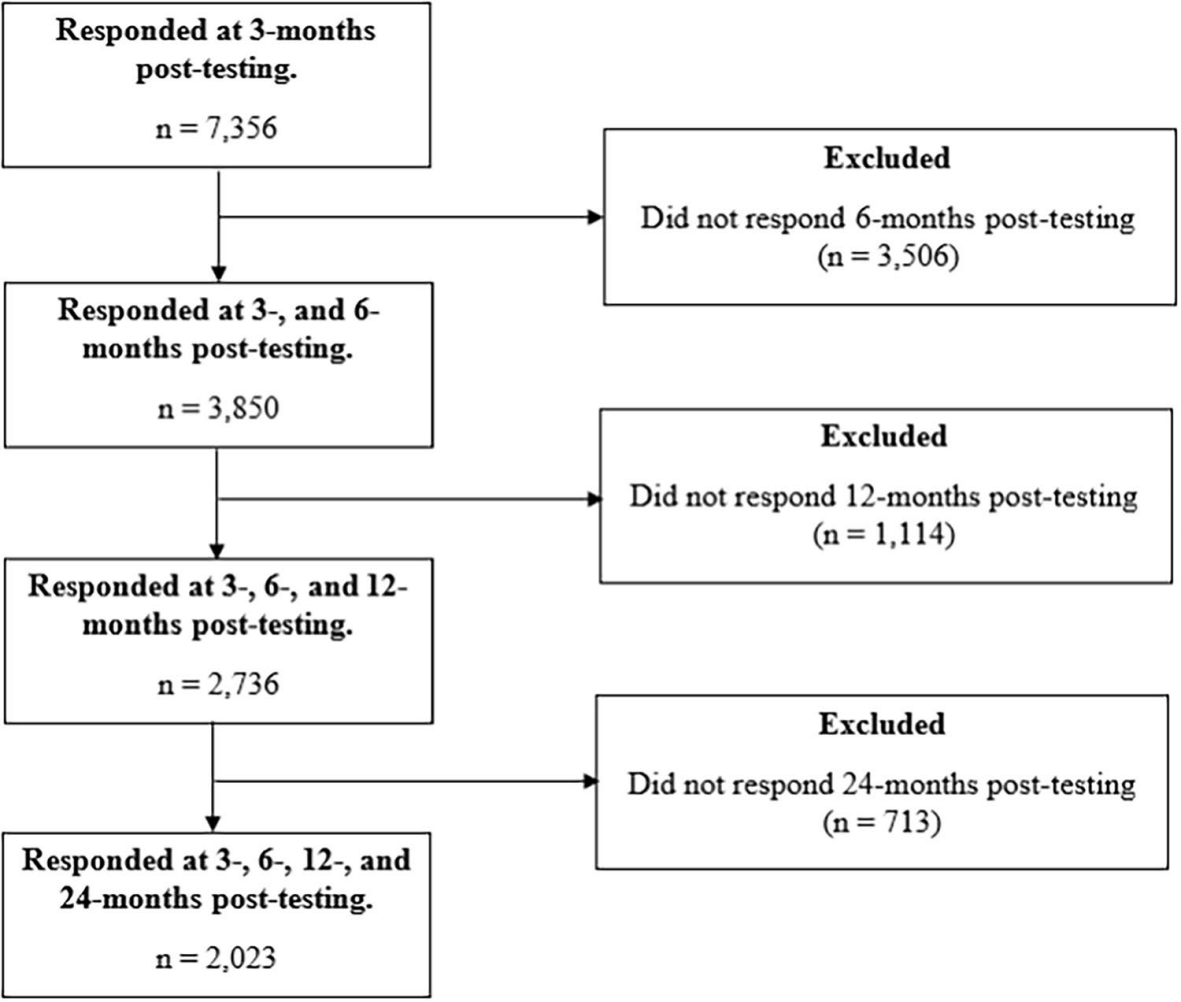
A flowchart of participants from enrolment into the CLoCk study to analytical sample under consideration

The final analytic sample consisted of 2023 CYP who returned their 3-, 6-, 12-, and 24-month questionnaires. All participants provided written informed consent to participate in the study. Figure 1 presents the exclusions made to the dataset for the final analytic sample.

The study was approved by Yorkshire and The Humber—South Yorkshire Research Ethics Committee (REC reference: 21/YH/0060; IRAS project ID: 293495).

### Study measures

We dichotomised participants’ age at time of PCR-testing into two groups, 11–14 and 15–17, based on English education key stage groups. Sex at birth was reported as male/female. The Index of Multiple Deprivation (IMD), derived from the adolescent’s lower super output area (a small local area level-based geographical hierarchy), was used as a proxy for socioeconomic status. We used IMD quintiles from most (quintile 1) to least (quintile 5) deprived. At enrolment, participants reported their ethnicity (Asian/Asian British; Black/African/Caribbean; Mixed; Other; Prefer Not to Say; Other), and whether they had an Education Health and Care Plan (EHCP), which indicates a need for extra learning support at school including special educational needs and learning disabilities [29]. Additionally, participants were asked to rate their mental and physical health before their test on a five-point scale (Very Poor; Poor; Okay; Good; Very Good) and indicate whether they engaged in psychological therapies prior to the pandemic.

At all data collection sweeps, the questionnaire included the self-reported Strengths and Difficulties Questionnaire (SDQ) [30, 31], a globally recognised validated mental health and wellbeing measure. The SDQ consists of 25 items which enquire about the young person’s emotional and behavioural attributes which form five subscales. Four of the subscales are summed to produce a total difficulties score ranging from 0 to 40. Higher scores indicate elevated mental health difficulties, with scores above 17 identifying individuals with difficulties in the top 10% of the population, scores above 15 indicating individuals in the top 20%, and scores up to 14 reflecting difficulties seen in 80% of the population [31]. The impact supplement assesses the impact of the emotional and behavioural difficulties on the child’s life, including whether their difficulties interfere with their home life and friendships. The impact score generated ranges from 0 to 10, with scores above 3 reflecting emotional and behaviour difficulties impacts in the top 10% of the population. Given the SDQ’s reliability, validity, and widespread use in large, nationwide, epidemiological studies [32], this study utilised the total difficulties and impact score as its mental health symptoms outcome measures.

### Potential confounding factors

Potential confounding factors were identified a-priori and consisted of (1) sociodemographic factors (age group, sex, ethnicity, and IMD); and (2) clinical factors (SARS-CoV-2 test result at study recruitment, prior physical health, prior mental health, EHCP status, and prior psychological therapies).

### Statistical analysis

All analyses were conducted in STATA v.18.0. Firstly, to see if there were differences between those included in our sample (participants who returned questionnaires at 3-, 6-, 12-, and 24-months) and those excluded, we examined the pre-pandemic characteristics of the two groups using Chi-square and Analysis of Variance (ANOVA) tests with Bonferroni correction (Table 1). We compared the pre-pandemic characteristics and SDQ total difficulties and impact scores of participants who self-reported pre-existing eating problems to those who reported no pre-existing eating problems (Table 2).

**Table 1:** Pre-pandemic characteristics of (i) participants who enrolled 3-months post-testing (ii) participants excluded from the analytic sample and (iii) participants included in the analytical sample

	3-Month sample (*N* = 7356)	Excluded sample (*N* = 5333)	Included sample (*N* = 2023)	*p*-value
			Response rate: 27.50%	
*Pre-existing eating problems*				0.115
No	6406 (87.09)	4624 (86.71)	1782 (88.09)	
Yes	950 (12.91)	709 (13.29)	241 (11.91)	
*Age (years)*				0.119
11–14	3129 (42.54)	2298 (43.09)	831 (41.08)	
15–17	4227 (57.46)	3035 (56.91)	1192 (58.92)	
*Sex at birth*				< 0.001
Male	2728 (37.09)	2074 (38.89)	654 (32.33)	
Female	4628 (62.91)	3259 (61.11)	1369 (67.67)	
*Ethnicity*				0.021
White	5421 (73.69)	3884 (72.83)	1.537 (75.98)	
Asian/Asian British	1111 (15.10)	820 (15.38)	291 (14.38)	
Black/African/Caribbean	279 (3.79)	217 (4.07)	62 (3.06)	
Mixed	367 (4.99)	269 (5.04)	98 (4.84)	
Other	125 (1.70)	98 (1.84)	27 (1.33)	
Prefer not to say	53 (0.72)	45 (0.84)	8 (0.40)	
*IMD quintile*				< 0.001
1 (most deprived)	1514 (20.58)	1170 (21.94)	344 (17.00)	
2	1462 (19.87)	1071 (20.08)	291 (19.33)	
3	1415 (19.24)	1029 (19.29)	386 (19.08)	
4	1419 (19.29)	1001 (18.77)	418 (20.66)	
5 (least deprived)	1546 (21.02)	1062 (19.91)	484 (23.92)	
*SARS-CoV-2 status at enrolment*				0.119
Negative	4035 (54.75)	2955 (55.41)	1080 (53.39)	
Positive	3321 (45.15)	2378 (44.59)	943 (46.61)	
*Prior physical health*				0.548
Very good/good	5616 (76.35)	4054 (76.82)	1562 (77.21)	
Ok	1582 (21.51)	1164 (21.83)	418 (20.66)	
Poor/Very poor	158 (2.15)	115 (2.16)	43 (2.13)	
*Prior mental health*				0.937
Very good/good	4558 (61.96)	3305 (61.97)	1253 (61.94)	
Ok	2110 (28.68)	1533 (28.75)	577 (28.52)	
Poor/very poor	688 (9.35)	495 (9.28)	193 (9.54)	
*EHCP*				< 0.001
No	6884 (93.58)	4956 (92.93)	1928 (95.30)	
Yes	472 (6.42)	377 (7.07)	95 (4.70)	
*Prior psychological therapies*				
No	6604 (89.78)	4783 (89.69)	1821 (90.01)	0.678
Yes	752 (10.22)	550 (10.31)	202 (9.99)	

3-month sample includes those who at 3-months post-testing filled in their first questionnaire. *p* value from chi-2 and ANOVA tests comparing included and excluded participants

*IMD *index of multiple deprivation, *EHCP *education, health and care plan

**Table 2:** Pre-pandemic characteristics, SDQ total difficulties and impact scores by pre-existing eating problems status (*N* = 2023)

	Pre-existing eating problems	No pre-existing eating problems
*Variable*		
Category	*N* (%) or mean (SD)	*N* (%) or mean (SD)
*Age (years)*		
11–14	62 (23.73)	769 (43.15)
15–17	179 (74.27)	1013 (56.85)
*Sex at birth*		
Male	47 (19.50)	607 (34.06)
Female	194 (80.50)	1175 (65.94)
*Ethnicity*		
Asian/Asian British	37 (15.35)	254 (14.25)
Black/African/Caribbean	4 (1.66)	58 (3.25)
Mixed	12 (4.98)	86 (4.83)
Other	7 (2.90)	20 (1.12)
Prefer not to say	1 (0.41)	7 (0.39)
White	180 (74.69)	1357 (76.15)
*IMD quintile*		
1—most deprived	55 (22.82)	289 (16.22)
2	59 (24.48)	332 (18.63)
3	43 (17.84)	343 (19.25)
4	47 (19.50)	371 (20.82)
5—least deprived	37 (15.35)	447 (25.08)
*SARS-CoV-2 status at enrolment*		
Negative	126 (52.28)	954 (53.54)
Positive	115 (47.72)	828 (46.46)
*Prior physical health*		
Good/very good	122 (50.62)	1440 (80.81)
Okay	92 (38.17)	310 (17.40)
Poor/very poor	90 (37.34)	32 (1.80)
*Prior mental health*		
Good/very good	59 (24.48)	1194 (67.00)
Okay	92 (38.17)	485 (27.22)
Poor/very poor	90 (37.34)	108 (5.78)
*EHCP*		
No	217 (90.04)	1711 (96.02)
Yes	24 (9.96)	71 (3.98)
*Prior psychological therapies*		
No	168 (69.71)	1653 (92.76)
*Mean SDQ total difficulties*		
3-months post-testing	17.21 (6.46)	10.24 (5.84)
6-months post-testing	17.41 (6.59)	10.66 (6.01)
12-months post-testing	17.63 (6.36)	11.31 (6.32)
24-months post-testing	17.21 (6.75)	11.77 (6.28)
*Mean SDQ impact (score)*		
3-months post-testing	2.46 (2.62)	0.66 (1.54)
6-months post-testing	2.49 (2.63)	0.71 (1.52)
12-months post-testing	2.54 (2.76)	0.79 (1.64)
24-months post-testing	2.35 (2.61)	0.83 (1.65)
Yes	73 (30.29)	129 (7.24)

*IMD* index of multiple deprivation, *EHCP* education health and care plan

Our first aim was to establish associations between eating problems and mental health of CYP at different points in the pandemic. Therefore, we conducted regression analyses assessing the relation between pre-existing eating problems and SDQ total difficulties and impact scores at each data collection sweep (Table 3).

**Table 3:** Associations between pre-existing eating problems status and unadjusted SDQ total difficulties scores at 3-, 6-, 12-, and 24-months post-testing (*N* = 2023)

SDQ total difficulties at	Mean difference in SDQ total score by eating problems status	SE	95% CI	*p* value
3-months post-testing	6.98	0.406	6.18–9.73	< 0.001
6-months post-testing	6.75	0.417	5.93–7.57	< 0.001
12-months post-testing	6.32	0.434	5.46–7.17	< 0.001
24-months post-testing	5.44	0.435	4.59–6.29	< 0.001

Results are presented as the unstandardised *b* coefficient, indicating the difference in mean SDQ total difficulties scores by pre-existing eating problems status

*SE *standard error, *CI *confidence interval

To establish whether CYP with pre-existing eating problems had differing SDQ total difficulties trajectories over time, from the CYP without eating problems, we performed longitudinal analyses using multi-level models estimated with restricted maximum likelihood. We constructed two models: in the first, we included an interaction term between pre-existing eating problems status and time to assess whether the effect of pre-existing eating problem status on SDQ mean total difficulties scores varied over the 2-year period as a fixed effect and included a participant-wise random intercept and random slope. In the second model, we additionally included the potential confounders listed above as fixed effects. Time was modelled as a metric variable calculated as the day of survey completion, which we centred at 0 on the first day of survey completion (13 April 2021), and then divided by 30 to aid interpretation on a month metric (see Table S1, Supplementary Information). We repeated these analyses with CYP’s SDQ impact scores as the outcome of interest (see Table S2, Supplementary Information).

## Results

### Sample characteristics

In our analytic sample of 2023, 241 self-reported eating problems prior to the pandemic (11.91%), compared to 1782 CYP who reported no eating problems prior (88.09%). Chi-square and ANOVA tests with Bonferroni correction were conducted. Table 1 presents the comparison of pre-pandemic characteristics between participants included and excluded from our sample. The only striking disparity is the smaller proportion of most deprived CYP in the participant sample analysed and, possibly related, fewer CYP with an EHCP.

Table 2 presents the sample’s pre-pandemic characteristics by baseline eating problems status. Chi-square and ANOVA tests with Bonferroni correction revealed that participants who self-reported having eating problems prior to the pandemic were significantly more likely to: be 15 to 17 years (74.27% compared to 56.85%), females (80.50% compared to 65.94%), live in more deprived areas (22.82% compared to 16.22%), report poorer physical (37.34% compared to 1.80%) and mental (37.34% compared to 5.78%) health prior to the pandemic, report having an EHCP (9.96% compared to 3.98%), and report having engaged in psychological therapies prior to the pandemic (30.29% compared to 7.24%).

Mann–Whitney *U* tests with Bonferroni correction revealed that older age, being female, poorer prior physical health, poorer prior mental health, EHCP status, and engagement in prior psychological therapies were associated with raised SDQ total difficulties scores at all time points (with the exception of age and SDQ total difficulties at 24-months post-testing).

### Associations between pre-existing eating problems and SDQ scores

Linear regression analyses showed that pre-existing eating problems were consistently associated with raised SDQ total difficulties (Table 3) and impact scores (Table 4) at all time points.

**Table 4:** Associations between pre-existing eating problems status and unadjusted SDQ impact scores at 3-, 6-, 12-, and 24-months post-testing (*N* = 2023)

SDQ impact at	Mean difference in SDQ impact score by pre-existing eating problems status	SE	95% CI	*p* value
3-months post-testing	1.80	0.118	1.57–2.03	< 0.001
6-months post-testing	1.78	0.117	1.55–2.01	< 0.001
12-months post-testing	1.75	0.125	1.50–2.00	< 0.001
24-months post-testing	1.52	0.124	1.28–1.76	< 0.001

Results are presented as the unstandardised *b* coefficient, indicating the difference in mean SDQ impact scores by pre-existing eating problems

*SE *standard error, *CI* confidence interval

### SDQ total difficulties scores over time by pre-existing eating problems status

**Fig. 2:**
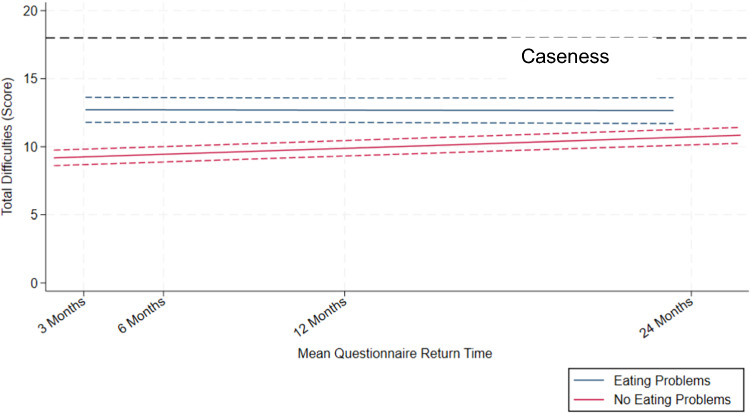
Mean SDQ total difficulties over time and 95% confidence intervals, by pre-existing eating problems status, adjusted for potential confounders. *Note* Time points reflect the mean questionnaire return time in months within each data collection sweep. Mean return time at 3 months = 9th May 2021; 6 months = 31st July 2021; 12 months = 3rd Feb 2022; 24 months = 21st Jan 2023. For the Eating Problems group, the first 3-month questionnaire was returned on 19th May 2021, and the last 24-month questionnaire was returned on 6th Jan 2023. For the No Eating Problems group, the first 3-month questionnaire was returned on 17th Apr 2021; and the last 24-month questionnaire was returned on 15th Mar 2023. The trajectory was modelled with an interaction between pre-existing eating problems status and time, *p* < 0.001. Scores were adjusted for initial SARS-CoV-2 result, age group, sex, IMD-5, ethnicity, prior physical and mental health, EHCP, and prior psychological therapies. CI = Confidence Interval, IMD = Index of Multiple Deprivation, EHCP = Education Health and Care Plan. Higher scores indicate more emotional and behavioural difficulties, with scores above 18 indicating scores in the top 10% of the population and guiding clinical caseness for mental health disorders

Figure 2 displays mean SDQ total difficulties over time and 95% confidence intervals, by pre-existing eating problems status, adjusted for potential confounders. Supplementary Table S1 presents the results of the multi-level models of the rate of change in SDQ total difficulties scores over time by pre-existing eating problems status, unadjusted and adjusted for confounders. In the unadjusted model, on average, those with pre-existing eating problems had 7.06 (95% CI [6.27, 7.84]) higher total difficulties score points at the start compared to those without eating problems. The association between pre-existing eating problems and SDQ total difficulties varied over time, such that those with eating problems experienced a slower rate of change on the SDQ over time by − 0.075 per month (95% CI [− 0.10, − 0.05]).

After adjusting for potential confounders, including prior mental health, on average those with eating problems had 3.55 (95% CI [2.67, 4.23]) higher total difficulties score points at the start compared to those without eating problems. The association between baseline eating problem status and SDQ total difficulties varied over time, such that those with eating problems experienced a slower rate of change on the SDQ over time by − 0.074 per month (95% CI [− 0.10, − 0.05]).

There was no significant change in total difficulties scores over time from 3 months to 24 months for those with eating problems (− 0.003 per month, 95% CI [− 0.03, 0.02]), whereas those without eating problems experienced a small yet significant increase in their total difficulties scores over time (0.07 per month, 95% CI [0.06, 0.08]). Taken together, our results suggest that whilst those with pre-existing eating problems had higher total difficulties scores on average, those *without* eating problems experienced a slight worsening of their total difficulties scores. In contrast, total difficulties did not significantly change over the examined time period in those with pre-existing eating problems. Moreover, the majority of the sample did not have scores which meet the threshold for clinical caseness. A comparison of the SDQ total difficulties trajectories between CYP with and without eating problems is displayed in Fig. 2.

### SDQ impact scores over time by eating problems status

**Fig. 3:**
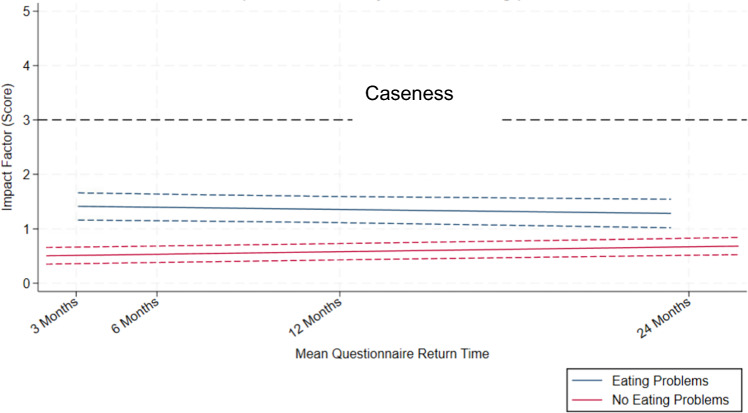
Mean SDQ impact scores over time and 95% confidence intervals, by pre-existing eating problems status, adjusted for potential confounders. *Note* Time points reflect the mean questionnaire return time in months within each data collection sweep. Mean return time at 3 months = 9th May 2021; 6 months = 31st July 2021; 12 months = 3rd Feb 2022; 24 months = 21st Jan 2023. For the Eating Problems group, the first 3-month questionnaire was returned on 19th May 2021, and the last 24-month questionnaire was returned on 6th Jan 2023. For the No Eating Problems group, the first 3-month questionnaire was returned on 17th Apr 2021; and the last 24-month questionnaire was returned on 15th Mar 2023. Trajectory was modelled with an interaction between pre-existing eating problems status and time, *p* < 0.001. Scores were adjusted for SARS-CoV-2 result, age group, sex, IMD-5, ethnicity, prior physical and mental health, EHCP, and prior psychological therapies. CI = Confidence Interval, IMD = Index of Multiple Deprivation, EHCP = Education Health and Care Plan. Higher scores indicate more impact of their emotional and behavioural difficulties with scores above 3 indicating impact in the top 10% of the population

Comparison of the SDQ impact trajectories between CYP with and without eating problems is displayed in Fig. 3. Supplementary Table S2 presents the results of the multi-level models of the rate of change in SDQ impact scores over time by pre-existing eating problems, unadjusted and adjusted for confounders. In the unadjusted model, on average, those with pre-existing eating problems had 1.84 (95% CI [1.62, 2.05]) higher impact score points at the start of the trajectory compared to those without eating problems. The association between pre-existing eating problems status and SDQ impact scores varied over time, such that those with eating problems experienced a slower rate of change on the SDQ impact score over time by − 0.0144 per month (95% CI [− 0.02, − 0.005]).

After adjusting for confounders, including prior mental health, those with eating problems had 0.91 (95%CI [0.71, 1.12]) higher SDQ impact score points at the start of the trajectory compared to those without eating problems. The association between eating problems status and SDQ impact scores varied over time, such that those with eating problems experienced a slower rate of change over time by − 0.0142 per month (95% CI [− 0.02, − 0.005]).

Moreover, the majority of the sample did not have impact scores which meet the threshold for clinical caseness.

## Discussion

The study sought first to determine associations between pre-existing eating problems and emotional and behavioural difficulties over a 24-month period in CYP. There was evidence of univariate associations between eating problems status and SDQ total difficulties and impact scores at all time points, such that those with pre-existing eating problems had elevated emotional and behavioural difficulties at all time points compared to those without. This difference remained, albeit diminished, when controlling for confounding variables such as prior mental health. The second aim of the study was to compare mental health trajectories of those with and without self-reported eating problems prior to the pandemic. There was a statistically significant difference between the mental health trajectories of those with and without eating problems. Those with pre-existing eating problems had stable total difficulties and impact scores over time, whilst those without eating problems had increased total difficulties and impact scores over the 2 years.

We expected CYP with pre-existing eating problems to have been especially vulnerable to mental health difficulties during the pandemic. This was based on previous literature [13, 14, 25] and because the pandemic created a context in which there was unpredictability, with concerns over the spread of the coronavirus as well as regarding school openings and closures. For CYP with eating problems, perceptions of uncertainty can have a negative impact on mental health [7]. Thus, we considered that the pandemic would have particularly negatively impacted the mental health of these CYP. Moreover, CYP with eating disorders report the importance of a strong support network especially in their recovery [33]. However, the social restrictions during the pandemic limited the extent to which CYP could interact with peers in person, and many people with eating problems reported in a survey that they had difficulties with feelings of social isolation and reduced access to their social support networks during the pandemic [18]. These individuals perceived their relationships with their peers as vital for their eating disorder recovery and feeling a loss of access to them had an adverse effect on their motivation for recovery, consequently continuing to engage in disordered eating behaviours.

However, our large cohort study with a longitudinal design revealed that those with pre-existing eating problems did not experience a significant change in their mental health scores over time, showing stable total difficulties and impact scores on the SDQ. In contrast, those *without* eating problems had a small increase in total and impact difficulties over time. This finding was surprising since we expected CYP with pre-existing eating problems to have been especially vulnerable to emotional and behavioural difficulties during the pandemic. CYP with eating disorders tend to have difficulties with their social functioning above and beyond the difficulties that are typically experienced by CYP without eating disorders [34]. The social restrictions imposed by the pandemic may have had less direct impact on the social functioning of these CYP. However, caution should be taken when concluding the impact of the pandemic on their mental health.

Whilst the social distancing measures likely decreased the social interactions of CYP with their peers, an important factor for eating disorder recovery [15], the social distancing measures also likely reduced interpersonal triggers [17]. Body-, eating-, and exercise-related social comparisons are related to body dissatisfaction and eating behaviours in women with eating disorders and disordered eating [35]. Thus, the social distancing measures likely reduced face-to-face body-based social comparisons, which may have served as a protective factor for CYP with eating problems. It is also important to note that the current study mapped the average changes in mental health over time which may conceal many different individual outcomes over time. For example, Soneson et al. [36] analysed the mental health and wellbeing of CYP aged 8–18 years from the OxWell Student Survey, reporting that 33% of CYP had improved mental wellbeing during the first lockdown in England [36].

CYP with eating disorders tend to be at higher risk for mental health problems, with high rates of comorbidity with other psychiatric conditions, including depressive and anxiety disorders [37]. Moreover, other psychiatric conditions, such as depression, are partly characterised by altered eating behaviours, and appetite and weight dysregulation [1]. Other research has suggested that disordered eating behaviours are comorbid with depressive disorders [38]. Thus, our finding that those with eating problems had raised SDQ total difficulties and impact scores relative to those without eating problems at all time points, yet stable trajectories, is not surprising. It is important to note that participants were not directly asked whether they had an eating disorder diagnosis or another mental health condition, such as depression. Nevertheless, the SDQ does not have any eating disorder symptom questions. The increased levels of emotional and behavioural difficulties on the SDQ by the CYP who reported eating problems suggest that they have more additional mental health difficulties. Despite this, the mean scores on the SDQ of the CYP who reported pre-existing eating problems did not meet the threshold for clinical caseness which was a positive finding.

### Strengths and limitations

The CLoCk study is the largest, national matched cohort study which has investigated the impact of the pandemic on the health of CYP. Our study provides valuable insight into the mental health outcomes over time of CYP with eating problems, benefitting from the large sample and clear sample frame, as well as longitudinal follow-up and use of a widely used, validated mental health measure. We were able to account for pre-pandemic demographic and clinical factors that could potentially confound the association between eating problems and mental health, such as age, sex, area-level deprivation, and ethnicity, which studies have previously identified as vulnerability factors for mental health difficulties during the pandemic [11]. However, the study also had a number of limitations. The distinction between participants with and without eating problems was based on a single item. Standardised self-report questionnaires, such as the Eating Disorder Examination Questionnaire (EDE-Q), could have been used to capture a broader range of eating problems. The use of a single item prevented differentiating between participants with eating problems as a primary issue and those for whom eating difficulties may have been secondary to other conditions. There is also a risk of recall bias since eating problems and symptoms were self-reported. For example, CYP self-reported their physical and mental health condition prior to the pandemic with no objective measure used. Participants were being asked about pre-pandemic eating problems at the time of enrolment (April–June 2021) when the pandemic began in March 2020. No data during the first year of the pandemic were collected. Thus, it could have been the case that children/adolescents with pre-existing eating problems showed a high increase in mental health problems during the first pandemic year which then remained stable during the second and third year. However, this could not be considered in the present study. It is also the case that the small increase in mental health problems in individuals without pre-existing eating problems could be attributed to a simple age effect, as the level of emotional problems increases in older adolescents in general [39]. Moreover, whether CYP had an Education Health and Care Plan (EHCP) was used as a proxy for special educational needs (SEN) since there was no clinical assessment. An EHCP is a legal document which outlines the individual’s special educational needs and the support they need. However, there may be CYP receiving SEN support without an EHCP, or with needs that are not as visible or severe. Indeed, in 2023, there were an estimated 1.2 million CYP who were receiving support for their SEN without an EHCP [40]. Moreover, we used the Index of Multiple Deprivation (IMD) as a proxy for socioeconomic status since individual level measures of socioeconomic status (e.g. parental income) were not measured. Whilst the IMD indicates deprivation at the area level, those who live in non-deprived areas may have been struggling financially, conversely, those in deprived areas may not be struggling. This is important since the financial burden on families during the pandemic was especially prominent in low-income families and this had a negative impact on the children’s mental health [41]. Future research may build upon our findings and attempt to explore the underlying mechanisms for these differences. For example, future studies could implement a mixed-methods approach incorporating both quantitative and qualitative measures of mental health outcomes to gain a deeper understanding of the mental health experiences of CYP during the pandemic. The CLoCk study has utilised both quantitative and qualitative methods, including semi-structured interviews to disentangle the impact of long COVID on young people [42], although this research did not focus on the mental health of CYP during the pandemic. Rather, quantitative and qualitative components in tandem would provide an integrated set of evidence addressing the question of the mental health impact of the pandemic for CYP [43]. The longitudinal analysis provides useful insight into the mental health experiences of CYP during the pandemic, a period of uncertainty and social and functional changes, in conjunction with the hormonal and cognitive changes during adolescence. Moreover, the follow-up period of 24 months provides insight into the mental health changes CYP experienced as they emerged from the pandemic, which introduced challenges such as adjusting to schools re-opening, and for the CYP who may now be transitioning to higher education, moving out of their homes and becoming more independent. Further understanding of how CYP have emerged from the pandemic necessitates longer-term follow-up on these CYP. Alternatively, our understanding of how CYP transitioned from the pandemic as restrictions were lifted may be facilitated by qualitative exploration.

## Conclusion

The present study found a significant difference between the mental health over time of CYP with and without disordered eating prior to the pandemic. There were more emotional and behavioural difficulties and impact at all time points amongst those with eating problems versus those without, but these did not get worse, encouragingly providing evidence of some resilience to the effects of the pandemic and its aftermath in this population.

### What is already known on this subject?

Eating disorders are a major public health concern and presentations have remained high since the COVID-19 pandemic.

CYP with eating disorders may be particularly susceptible to pandemic-related challenges, including social and functional restrictions.

### What this study adds?

Our large cohort study with a longitudinal design revealed that those with pre-existing eating problems did not experience a significant change in their mental health scores over time, showing stable total difficulties and impact scores on the SDQ.

Understanding how eating difficulties relate to mental health symptomology over time has implications for service planning.

## Supplementary Information

Below is the link to the electronic supplementary material.

Supplementary file 1 (DOCX 26 KB)

## Data Availability

No datasets were generated or analysed during the current study.
