# Supplementary material for: Mental health of children and young people with pre-existing eating problems during the COVID-19 pandemic
Source: Eat Weight Disord. 2025 Sep 29;30(1):77. doi: 10.1007/s40519-025-01788-3 (PMC12479571; doi:10.1007/s40519-025-01788-3)
Supplement: Supplementary file 1 — (DOCX 26 KB) [file 40519_2025_1788_MOESM1_ESM.docx]

**Supplementary Table 1. A table presenting the results from the unadjusted and adjusted multi-level models of SDQ total difficulties scores over time by baseline eating problems status (N = 2,023).**

| **Variable** | **Mean Difference in SDQ total score** | **SE** | **95% CI** | ***p* value** |
| --- | --- | --- | --- | --- |
| **Model 1: Unadjusted** |  |  |  |  |
| **Time (month)** | 0.0714 | 0.005 | 0.06 to 0.08 | *< .001* |
| **Eating Problems** [ref: No Eating Problems] | 7.06 | 0.399 | 6.27 to 7.84 | *< .001* |
| **Difference in rate of change in mean SDQ (per month) between those with and without Eating Problems** | -0.0747 | 0.0139 | -0.10 to -0.05 | *< .001* |
| **Mean SDQ at start of trajectory** | 10.34 | 0.138 | 10.07 to 10.61 | *< .001* |
| **Model 2: Adjusted for potential confounders*** |  |  |  |  |
| **Time (month)** | 0.0715 | 0.00482 | 0.06 to 0.08 | *< .001* |
| **Eating Problems** [ref: No Eating Problems] | 3.55 | 0.385 | 2.79 to 4.30 | *< .001* |
| **Difference in rate of change in mean SDQ (per month) between those with and without Eating Problems** | -0.0744 | 0.0139 | -0.10 to -0.05 | *< .001* |
| **Mean SDQ at start of trajectory** | 9.17 | 0.292 | 8.60 to 9.74 | *< .001* |

*Note*. Results are presented as the unstandardised beta coefficients for the difference in mean SDQ total difficulties score (*b* per month), SE = Standard Error, CI = Confidence Intervals, ref = Reference Category. Potential confounders* age (11-14 years, 15-17 years), sex at birth (Female, Male), ethnicity (White, Asian/Asian British, Black/African/Caribbean, Mixed, Other, Prefer not to say), initial SARS-CoV-2 results (Negative, Positive), prior physical health (Good/Very Good, Okay, Poor/Very Poor), prior mental health (Good/Very Good, Okay, Poor/Very Poor), EHCP status (No, Yes), prior psychological therapies (No, Yes).

**Supplementary Table 2. A table presenting the results from the unadjusted and adjusted mixed-effects models of SDQ impact scores over time by baseline eating problems status (N = 2,023).**

| **Variable** | **Mean Difference in SDQ impact score** | **SE** | **95% CI** | ***p* value** |
| --- | --- | --- | --- | --- |
| **Model 1: Unadjusted** |  |  |  |  |
| **Time (month)** | 0.0077 | 0.00167 | 0.004 to 0.01 | *< .001* |
| **Eating Problems** [ref: No Eating Problems] | 1.84 | 0.110 | 1.62 to 2.05 | *< .001* |
| **Difference in rate of change in mean SDQ (per month) between those with and without Eating Problems** | -0.0144 | 0.00485 | -0.02 to -0.005 | *.003* |
| **Mean SDQ at start of trajectory** | 0.681 | 0.0380 | 0.61 to 0.75 | *< .001* |
| **Model 2: Adjusted for potential confounders*** |  |  |  |  |
| **Time (month)** | 0.0077 | 0.00167 | 0.004 to 0.01 | *< .001* |
| **Eating Problems** [ref: No Eating Problems] | 0.91 | 0.106 | 0.71 to 1.12 | *< .001* |
| **Difference in rate of change in mean SDQ (per month) between those with and without Eating Problems** | -0.0142 | 0.00485 | -0.02 to -0.005 | *.003* |
| **Mean SDQ at start of trajectory** | 0.505 | 0.0783 | 0.35 to 0.66 | *< .001* |

*Note*. Results are presented as the unstandardised beta coefficients for the difference in mean SDQ total difficulties score (*b* per month), SE = Standard Error, CI = Confidence Intervals, ref = Reference Category, Potential confounders* age (11-14 years, 15-17 years), sex at birth (Female, Male), ethnicity (White, Asian/Asian British, Black/African/Caribbean, Mixed, Other, Prefer not to say), initial SARS-CoV-2 results (Negative, Positive), prior physical health (Good/Very Good, Okay, Poor/Very Poor), prior mental health (Good/Very Good, Okay, Poor/Very Poor), EHCP status (No, Yes), prior psychological therapies (No, Yes).
